# Generation of novel high quality HMW-GS genes in two introgression lines of *Triticum aestivum*/*Agropyron elongatum*

**DOI:** 10.1186/1471-2148-7-76

**Published:** 2007-05-15

**Authors:** Shuwei Liu, Shuangyi Zhao, Fanguo Chen, Guangmin Xia

**Affiliations:** 1 School of Life Sciences, Shandong University, Jinan, 250100, PR China

## Abstract

**Background:**

High molecular weight glutenin subunits (HMW-GS) have been proved to be mostly correlated with the processing quality of common wheat (*Triticum aestivum*). But wheat cultivars have limited number of high quality HMW-GS. However, novel HMW-GS were found to be present in many wheat asymmetric somatic hybrid introgression lines of common wheat/*Agropyron elongatum*.

**Results:**

To exploit how these new subunits were generated, we isolated HMW-GS genes from two sib hybrid lines (II-12 and 11-4-6) and compared them with those from their parents. The result shows that two genes of hybrid (*H11-3-3 *and *H11-4-3*) are directly introgressed from the donor parent *Agropyron elongatum*; one hybrid gene (*H1Dx5*) comes from point mutation of a parental wheat gene (*1Dx2.1*); two other hybrid genes (*H1By8 *and *H1By16*) are likely resulting from unequal crossover or slippage of a parental wheat gene (*1By9.1*); and the sixth novel hybrid gene (*H1Dy12*) may come from recombination between two parental genes.

**Conclusion:**

Therefore, we demonstrate that novel HMW-GS genes can be rapidly created through asymmetric somatic hybridization in a manner similar with the evolution mechanism of these genes supposed before. We also described gene shuffling as a new mechanism of novel HMW-GS gene formation in hybrids. The results suggest that asymmetric somatic hybridization is an important approach for widening HMW-GS genebank of wheat quality improvement.

## Background

High molecular weight glutenin subunits are important component of wheat gluten proteins which are mainly responsible for dough viscosity and elasticity in wheat processing [1]. The HMW-GS accounts for up to about 12% of the total protein in the endosperm of common wheat (*Triticum aestivum *L.), while their allelic variation explains about 45% to 70% of the variation in bread making performance within European wheat cultivars [2-4]. Thus, their coding sequences are the prime candidates for molecular modification to enhance grain-processing quality [5].

The genes encoding the wheat HMW-GS are located on the long arms of the homoeologous group 1 chromosomes [6-9]. Each locus consists of two tightly linked genes, termed x- and y-type genes according to their different molecular weights. So far, the structural characteristics of more than ten HMW-GS alleles have been revealed by DNA sequencing [10-18]. All of these HMW-GS contain a central repetitive domain flanked by non-repetitive N- and C-terminal domains. The central domain of both the x- and the y-type subunits comprises hexapeptide and nonapeptide motifs, but the x-type subunits also contain tripeptide motifs. The presence of such a large repetitive central domain probably provides the basis for major and rapid structural changes via duplication and/or deletion of large segments as a result of unequal cross over [19].

The HMW-GS proteins are important for wheat processing quality; however, the number of HMW-GS alleles with good quality is very limited in wheat cultivars. So, peoples turn to wheat-related grasses for new good quality HMW-GS allele for wheat quality improvement. But many HMW-GS in wheat-related grasses are smaller than those of normal wheat [20-24], while the length of HMW-GS has been proved to be positively correlated with dough strength. Thus, the simple introgression or transformation of HMW-GS from grasses is not sufficient for the wheat quality breeding.

Asymmetric somatic hybridization between protoplast of common wheat (*Triticum aestivum *L cv. Jinan 177) and UV-irradiated protoplast of *Agropyron elongatum *can generate fertile introgression lines with superior agronomic traits, in particular the bread-making quality [25-27]. About 35% of the 175 somatic hybrids express novel high quality HMW-GS subunits not present in Jinan177 (1Bx7.1+1By9.1, 1Dx2.1+1Dy12.1) or both parents [28]. For example, line II-12 has good bread-making quality, and its HMW-GS profile is H1Ax2*, H1Bx13+H1By16, H1Dx5+H1Dy12 [26]. The identification of the origination of these new subunits will benefit the understanding of the process and guide us to identify and produce more new HMW-GS genes through the somatic hybridization. We speculated the possible formation process of novel genes previously by sequencing the *H1Bx13+H1By16 *and *H1Ay *genes of introgression line II-12 [26]. In this report, we isolated and sequenced all the HMW-GS genes of both II-12 and another sib introgression line, which also has a special HMW-GS profile different from Jinan177, and the origination of novel HMW-GS genes in hybrid wheat was discussed.

## Results and discussion

### SDS-PAGE profile of HMW-GS

**Figure 1:**
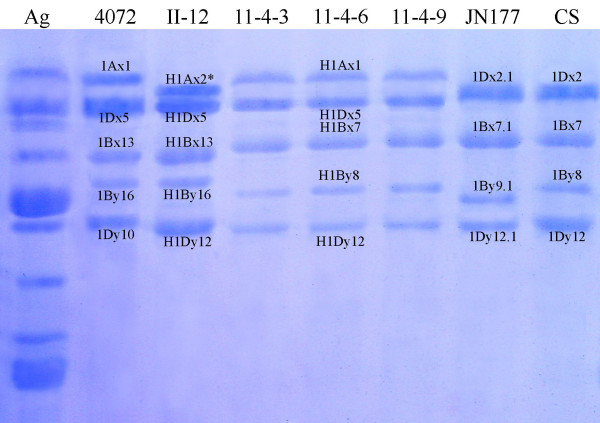
SDS-PAGE analysis of high-molecular-weight glutenin subunits (HMW-GS) of some common wheats and introgression lines. Seeds of *A. elongatum *contain nine subunits. The HMW-GS composition of different genotypes of common wheat is listed as following: JN177 is 1Bx7.1+1By9.1 and 1Dx2.1+1Dy12.1; CS (Chinese Spring) is 1Bx7+1By8 and 1Dx2+1Dy12; 4072 is 1Ax1, 1Bx13+1By16 and 1Dx5+1Dy10. The HMW-GS composition of hybrid II-12 and 11-4-6 are "H1Ax2*, H1Bx13+H1By16, H1Dx5+H1Dy12" and "H1Ax1, H1Bx7+H1By8, H1Dx5+H1Dy12" respectively.

The SDS-PAGE profile of HMW-GS showed that the parent wheat Jinan177 has the combination of "1Bx7.1+1By9.1; 1Dx2.1+1Dy12.1" while another parent *A. elongatum *contains nine HMW-GS in seeds; the HMW-GS composition of hybrid line II-12 and 11-4-6 appeared to be "H1Ax2*; H1Bx13+H1By16; H1Dx5+H1Dy12" and "H1Ax1; H1Bx7+H1By8; H1Dx5+H1Dy12" similar to bread wheat, respectively (Figure 1).

### HMW-GS genes from both parents and two introgression lines

**Figure 2:**
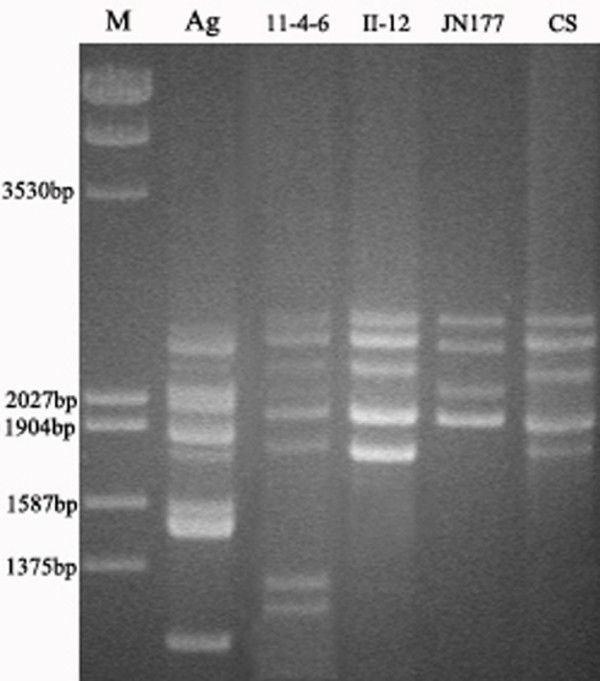
PCR profile of hybrids and parents DNA amplified with the degenerate primer pairs, CS DNA was used as control and M was Marker (*Eco*R I/*Hin*d III-digested λ DNA).

The amplified product of parent wheat Jinan177 and two introgression lines II-12 and 11-4-6 ranged from 1.8 kb to 2.5 kb, but two smaller fragments also appeared in the 11-4-6; the amplicon of another parent *A. elongatum *ranged from 1.1 kb to 2.4 kb (Figure 2). All the amplified products of both parents and the two hybrid lines were cloned and a lot of inserts with different sizes were obtained. After sequencing the terminal region of these inserts we ascertained the identity of these inserts. We selected some inserts to represent the coding region of subunits of parents and the two hybrids, then, we obtained their full length sequences by nested deletion. Through comparison of the mobility of proteins expressed in *E. coli *directed by some sequences with those of subunits in seeds and alignment of these sequences with those published HMW-GS alleles (date not shown) of common wheat, four sequences were designated *1Dx2.1 *(GenBank: DQ478570), *1Bx7.1 *(GenBank: DQ478571), *1By9.1 *(GenBank: DQ000162) and *1Dy12.1 *(GenBank: DQ000161) to represent the ORFs of 1Bx7.1+1By9.1 and 1Dx2.1+1Dy12.1 of parent Jinan177 respectively (Table 1); *H1Dx5 *(GenBank: DQ478572), *H1Dy12 *(GenBank: DQ478573) and *H1By8 *(GenBank: DQ646520) were designated as the coding sequences of H1Dx5+H1Dy12 of II-12 and H1By8 of hybrid 11-4-6, the character H in these names means that these sequences come from hybrid introgression lines. We also acquired two sequences from hybrid 11-4-6 which were different from any of the four HMW-GS alleles cloned from parent Jinan177 and we named them *H11-3-3 *(GenBank: DQ656419) and *H11-4-3 *(GenBank: DQ646521). However, both of them were very similar to *Aey1 *(GenBank: AY899822) and *Aex4 *(GenBank: DQ534448) cloned from *A. elongatum *respectively. The other two smaller fragments cloned in the amplicon of 11-4-6 were 1333 bp and 1260 bp, respectively. After sequence alignment, we found that they came from deletion of large fragments of *H1By8*. Therefore, we inferred that they were artifacts of PCR amplification and did not select them for further analyzes. There are no corresponding subunits of *H11-3-3 *and *H11-4-3 *in hybrid 11-4-6. The *H11-3-3 *contains in frame stop codon and frame shift mutation in its' ORF, therefore it can not express intact proteins in the seed. The reason why proteins encoded by *H11-4-3 *absent from seeds of 11-4-6 is unknown, we suggest that *H11-4-3 *was silenced in hybrids via DNA methylation because we have found that about 12% loci of the hybrids were hypermethylated using MSAP platform (Liu et al., personal communication). The other alleles cloned from *A. elongatum *were not shown in this paper. Amino acid sequences derived from all these sequences possessed the characteristic of HMW-GS of bread wheat, with a 21 amino acid residues signal peptide, N- and C-terminal conserved regions and a central repetitive region. The detailed structures of subunits derived from these sequences and some representative subunits of bread wheat were shown in Table 1.

**Table 1:** A summary of some of the properties of the primary structure of deduced peptides of hybrids and both parents and that of some representative subunits of bread wheat

Subunits	Accession number	Signal peptide	N-terminus domain	C-terminus domain	Repetitive domain	Total
1Dx2.1	DQ478570	21	89	42	687	839
H1Dx5	DQ478572	21	89	42	687	839
1Dx2	X03346	21	88	42	687	838
1Dx5	X12928	21	89	42	687	839
1Dy12.1	DQ000161	21	104	42	491	658
H1Dy12	DQ478573	21	104	42	491	658
Aey2	AY263343	21	104	42	491	658
1Dy12	X03041	21	104	42	493	660
1Bx7.1	DQ478571	21	81	42	645	789
H1Bx13	AY424400	21	81	42	651	795
1Bx7	X13927	21	81	42	645	789
1By9.1	DQ000162	21	104	42	538	705
H1By8	DQ646520	21	104	42	553	720
H1By16	AY263346	21	104	42	571	738
1By9	X61026	21	104	42	538	705
1By8	AY245797	21	104	42	553	720
H11-4-3	DQ646521	21	81	42	548	692
Aex4	DQ534448	21	81	42	548	692

### Novel HMW-GS genes from the introgression of donor to receptor

**Figure 3:**
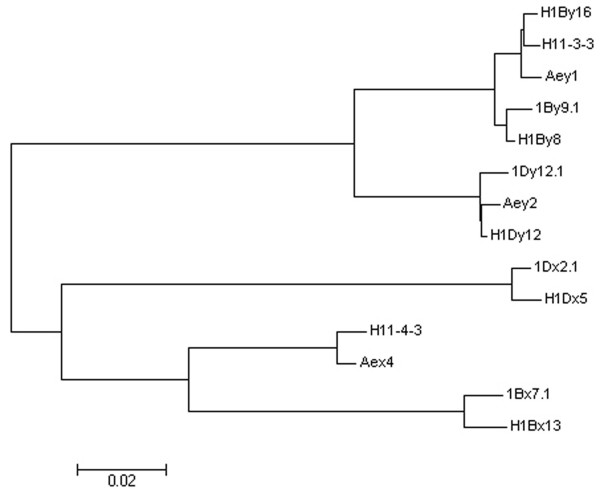
Phylogenetic analysis of HMW-GS genes cloned from hybrids and both parents. The Neighbor-Joining tree is constructed through MEGA program (Version 3.1).

*H11-3-3 *and *H11-4-3 *of hybrid wheat 11-4-6 displayed a high similarity with *Aey1 *and *Aex4 *of *A. elongatum*, but low similarity with all HMW-GS alleles of Jinan177 (Figure 3), which confirmed that these two sequences might be transferred from *A. elongatum *to hybrid wheat genome during somatic hybridization. GISH analysis of hybrid wheat genome in our lab revealed that different hybrid lines had different sites of translocation or insertion of chromatin [29,30]. Wang et al. [31] located introgression small-chromosome-segments of *A. elongatum *on hybrid wheat chromosomes 2AL, 1BL, 5BS, 1DL, 2DL and 6DS, using GISH/FISH/SSR analysis combined with karyotype data. However, there is no direct evidence concerning the hybrid introgression line contains gene from donor on the molecular level. The sequence data of these two genes provided further evidence that asymmetric somatic hybridization can transfer alien gene(s) from donor to the receptor genome, and the potential as a genetic/genomic tool for crop improvements.

### Novel HMW-GS gene present in hybrid via point mutation of parent gene(s)

**Figure 4:**
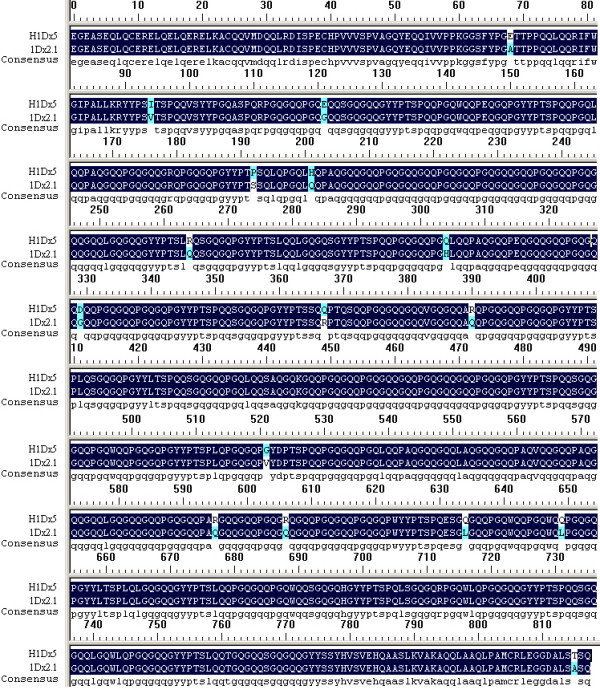
Comparison of mature peptide sequences deduced from DNA sequences of *H1Dx5 *and *1Dx2.1*.

Sequence alignment indicated that H1Dx5 was similar to 1Dx2.1 of parent Jinan177 with 16 amino acid substitutions (Figure 4) and we did not found any sequences similar to H1Dx5 from *A. elongtum*, so we confirmed that H1Dx5 of hybrid came from 1Dx2.1 of parent wheat via point mutation in the process of hybridization. Both H1Dx5 and 1Dx2.1 shows more similarity to 1Dx2 (GenBank: X03346) than 1Dx5 (GenBank: X12928) of bread wheat. However, they do not share the position 60 amino acid deletion characteristic of subunit 1Dx2. Bacterial expression of modified ORFs of *H1Dx5 *and *1Dx2.1 *confirmed that they are coding sequences of H1Dx5 and 1Dx2.1 respectively (data not shown). Although molecular weights of deduced amino acids of H1Dx5 and 1Dx2.1 are similar, the migration of these two subunits on SDS-PAGE is obviously different (Figure 1). The reason of such anomaly is not known. However, it has been reported that the migrations of allelic subunit pair 1Dx2/1Dx5 and 1Dy10/1Dy12 was anomalous, subunit 1Dx5 has a higher mobility than the smaller allelic 1Dx2, and similarly, 1Dy10 has a lower mobility than the larger allelic 1Dy12 [32]. Through Urea SDS-PAGE and making chimeric genes, Goldsbrough et al. [33] found that the anomaly of migration on SDS-PAGE between 1Dy10 and 1Dy12 might be resulted from conformational differences induced by six amino acid substitution between the two subunits. Urea SDS-PAGE of recovered H1Dx5 and 1Dx2.1 proteins showed that the two subunits appeared nearly the same electrophoretic mobility in presence of urea where secondary structure of these proteins is broken down and the proteins are separated according to their relative molecular weight (data not shown). Bread making-quality analysis showed that hybrid-derived H1Dx5 subunit was related to good grain-processing quality, while 1Dx2.1 subunit was correlated with bad ones [27]. Therefore, we speculated that the same conformational difference was responsible for both the observed grain-processing quality differences and the anomalous electrophoretic behavior.

### Hybrid novel gene from allelic variation of parents via unequal crossover or slippage

**Figure 5:**
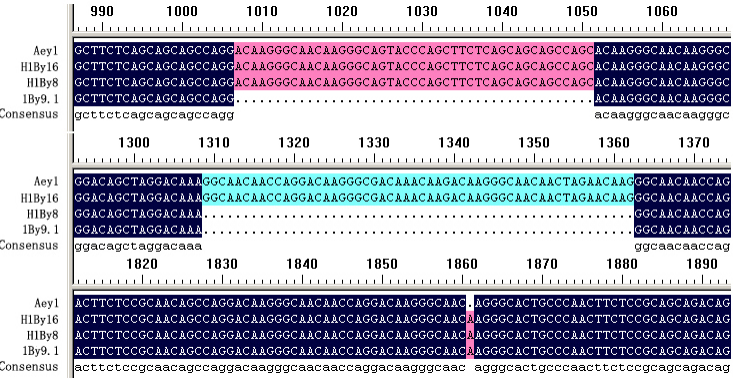
Comparison of part DNA sequences from repetitive domain of three *1By *alleles and a y-type allele *Aey1 *of *A. elongatum*. *H1By16 *and *H1By8 *is the coding sequence of novel subunit of hybrid; *1By9.1 *is the coding sequence of 1By9.1 subunit of common wheat Jinan177.

Sequence alignment indicated that *H1By16 *(GenBank: AY263346, cloned by Feng et al. [26]) and *H1By8 *were very similar with *1By9.1 *except the addition of repeat motifs and few SNPs. *H1By8 *contains an additional block of repeat than *1By9.1 *while *H1By16 *contains another repeat than *H1By8 *(Figure 5). Although *H1By16 *shows more similarity with *Aey1*, the latter has a single base deletion which results in a frame shift mutation, so *H1By16 *might not be acquired from *A. elongatum*. *H1By8 *and *H1By16 *should be formed by unequal crossover or slippage of *1By9.1 *to duplicate block of repeats. This is coincident with variation of SSR loci in hybrids. There present obvious variation in band pattern of some SSR loci between hybrid and parents using 173 primer pairs. Sequencing of some novel bands indicates that the variation of band pattern is mainly due to the addition or deletion of one or more repeat motifs (data not shown). Addition or deletion of repeat motifs maybe an effective mechanism of variation happened in sequences that contain many repeat motifs in them.

### Shuffling of parents genes to produce novel chimeric HMW-GS genes

**Figure 6:**
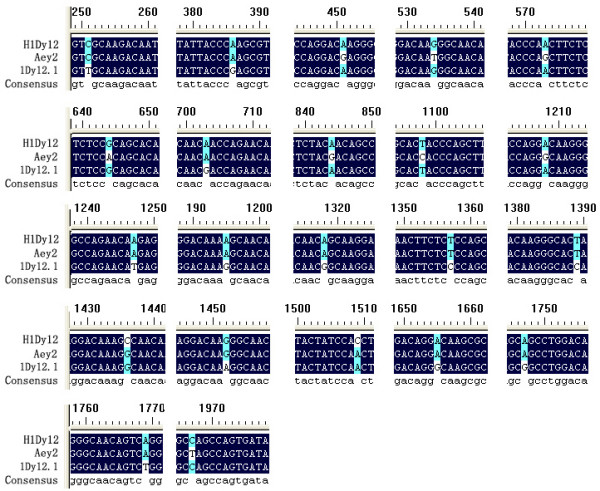
Comparison of peptide sequences deduced from three 1980 bp DNA sequences: *H1Dy12*, *Aey2 *and *1Dy12.1*. Seven of 10 discrepant bases before position 1210 are the same between *1Dy12.1 *and *H1Dy12*, while 9 of another 12 discrepant bases after position 1210 are the same between *H1Dy12 *and *Aey2*.

**Figure 7:**
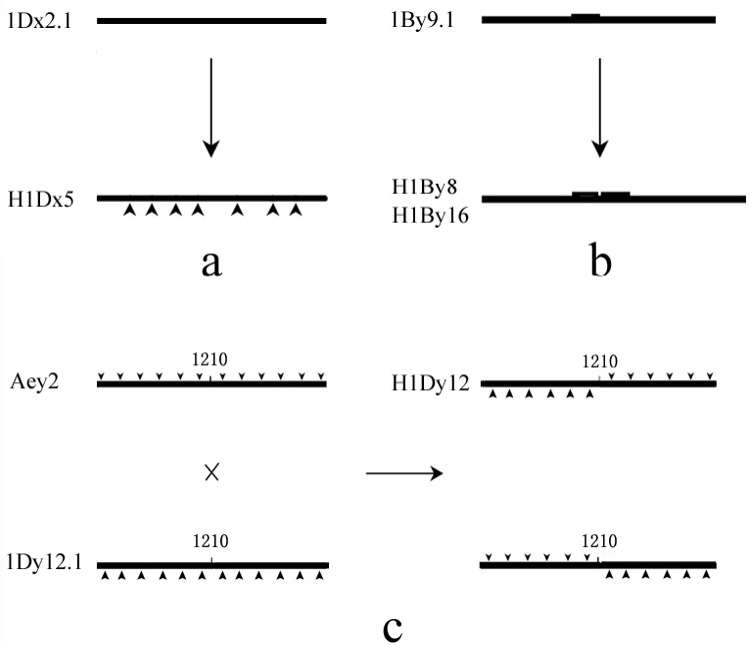
Sketch map of possible pathway through which novel hybrid HMW-GS genes come from, a): *H1Dx5 *come from *1Dx2.1 *via single base mutation, the arrow means the SNPs. b): *H1By8 *and *H1By16 *of hybrid generate from duplication of a block of repeats of *1By9.1*. c): *H1Dy12 *of hybrid engender from recombination between *Aey2 *of *A. elongatum *and *1Dy12.1 *of parent wheat Jinan177.

The coding sequence of H1Dy12 shows very high similarity with that of 1Dy12.1, with only a few SNPs, both of them are 1980 bp in length. The 1980 bp's long y-type sequence cloned from *A. elongatum *(*Aey2*, GenBank: AY263343[21]) makes us a little doubt about the origin of *H1Dy12 *of hybrid II-12. The three highly homologous 1980 bp sequences (*1Dy12.1*, *H1Dy12 *and *Aey2*) contain 22 different SNPs in all. Sequence alignment reveals that 7 of 10 discrepant bases before position 1210 are the same between *1Dy12.1 *and *H1Dy12*, while 9 of another 12 discrepant bases after position 1210 are the same between *H1Dy12 *and *Aey2 *(Figure 6). Therefore we conclude that the *H1Dy12 *of hybrid is likely a chimeric gene generated by recombination between *1Dy12.1 *and *Aey2 *(Figure 7). This is coincident with LMW-GS genes found in another sib hybrid wheat introgression line 7-4-1-2 in our lab. Chen et al. cloned and sequenced LMW-GS genes from the line and both parents, and found some chimeric genes (Chen, personal communication). As for the 6 bases that were different between *H1Dy12 *and corresponding parent genes, they might be the result of single base mutation during the process of UV-irradiation to the donor in the asymmetric somatic hybridization of the parent wheat and *A. elongatum *[25] and long-term tissue culture of parents/hybrids before or after the cell fusion, just like that happened in H1Dx5.

### Comparison of new gene generation from intergeneric hybridization with natural HMW-GS gene evolution

From evolutionary view, it was suggested that the evolutionary origin of HMW-GS involved a same ancestor [8,12,14,34]. Anderson and Greene [14] proposed a simplest mechanism about the sequence evolution of HMW-GS genes, which include: (a) single base changes (b) deletions or additions within a repeat (c) single repeat changes (d) deletions or duplications of blocks of repeats. Through cloning and sequencing of HMW-GS genes of the sib hybrid lines and both parents, we found that asymmetric somatic hybridization could not only transfer donor genes to the hybrid genome, but also lead to the emergence of new genes (Figure 7). The formation of some novel hybrid genes was inosculated with the mechanism mentioned by Anderson and Greene [14]. Moreover, we found gene shuffling in hybrid can also produce novel HMW-GS gene, just like that happened in the origination of some novel genes in *Drosophila *[35-37]. Thus, the novel HMW-GS and combinations generated in the sib asymmetric somatic hybrid lines from a same fusion cell are similar to the natural emergence of new HMW-GS but in less time.

### The novel HMW-GS with wheat quality breeding

In our investigation, many hybrid-derived novel HMW-GS show correlation with good grain-processing quality, such as H1Bx13+H1By16, H1Dx5+H1Dy12 and H1By8 subunits (GenBank: AY424400, AY263346, DQ478572, DQ478573 and DQ646520 respectively) in II-12 and other sib lines [27]. We are using them in gene transformation and marker-assistant quality breeding of common wheat, and a lot of germplasms and strains with high qualities have been produced from them. Therefore, asymmetric somatic hybridization between common wheat and relative intergeneric grasses can be an important tool for wheat quality breading.

## Conclusion

Through sequencing and comparing HMW-GS genes from introgression lines of wheat/*A. elongatum *and both parents, we confirmed that genes of *A. elongatum *had been introgressed into wheat in asymmetric somatic hybridization. It is significant that some novel HMW-GS genes with good processing quality could be generated in the introgression lines via the asymmetric somatic hybridization. The formation of these novel genes showed similarity to the evolutionary mechanism of natural HMW-GS genes proposed before, except that gene shuffling was found to be a special mode to form novel chimeric genes in the hybrid lines. The result suggests that asymmetric somatic hybridization is a unique pathway to produce novel genes that can be used for wheat quality improvement.

## Methods

### Plant materials

*Agropyron elongatum *(Host) Nevski [*Thinopyrum ponticum *2n = 10x = 70], common wheat Jinan 177 (*Triticum aestivum *L. 2n = 42), the introgression self-fertilized lines II-12 and 11-4 originated from a single fusion cell in the intergeneric asymmetric somatic hybridization between protoplasts of *T. aestivum *cv. Jinan177 and UV-irradiated protoplasts from *A. elongatum *were used in the experiments. II-12 is genetically stable and has the HMW-GS profile of H1Ax2*, H1Bx13+H1By16, H1Dx5+H1Dy12. Line 11-4 segregates in F_2 _generation with different HMW-GS, so a single seed selection (11-4-6) with the profile H1Ax1; H1Bx7+H1By8 and H1Dx5+H1Dy12 similar to common wheat was used to represent this genotype. Common wheat Chinese Spring (1Bx7+1By8, 1Dx2+1Dy12) and wheat cultivar 4072 (1Ax1, 1Bx13+1By16, 1Dx5+1Dy10), used for control, was kindly offered by Shandong Academy of Agricultural Sciences, Jinan, China.

### SDS-PAGE analysis of HMW-GS

HMW-GS of parent Jinan177 and hybrid lines II-12 as well as 11-4 was extracted from embryo-less half grains while those of *A. elongatum*, Chinese Spring and 4072 was extracted from the whole seeds as described by Mackie et al. [38]. SDS-PAGE was conducted following the procedures described by Feng et al. [20].

### Cloning and characterizing of HMW-GS genes

The seedlings of *A. elongatum *and that of embryo-carrying half seeds of Jinan177 and both introgression lines were grown in darkness for 14 days at 23°C. Genomic DNA was extracted from these seedlings using the CTAB method according to Murray and Thompson [39]. As the HMW-GS genes are free of introns, genomic DNA is used as template for PCR amplification of the entire coding region. In order to amplify the complete HMW-GS ORF of the two introgression lines and the two parents, a pair of degenerate primers was designed based on published DNA sequences. These were P1 (5'-ATGGCTAAGCGGC/TTA/GGTCCTCTTTG-3') and P2 (5'-CTATCACTGGCTA/GGCCGACAATGCG-3'). P1 includes the HMW-GS ORF start codon, and P2 has the two conserved tandem stop codons. The PCR required LA Taq polymerase (TaKaRa Biotechnology) with a GC buffer for GC-rich template. The amplification profile was one cycle at 95°C for 5 min, followed by 28 cycles of 94°C for 40 s, 68°C for 4 min, and a final extension step at 72°C for 7 min. The amplified product were recovered from 1.0% agarose gels, cloned into the pUCm-T vector, and transformed into *E. coli *DH10B competent cells. To obtain a full-length sequence, a series of five to six subclones were prepared using the nested deletion method of Sambrook et al. [40]. Sequencing was performed commercially (Invitrogen). Both amplification and cloning were repeated at least three times to minimize the possibility of errors present in amplification and sequencing. Sequence analyses were performed with the help of MEGA (Version 3.1) and programs from the NCBI and EBI networks.

## Authors' contributions

Shuwei Liu made substantial contribution to acquisition and analysis of data and drafting of the manuscript.

Shuangyi Zhao and Fanguo Chen made contribution to design of the work and analysis of the data. Both of them reviewed the manuscript.

Guangmin Xia has made substantial contribution to design of the work and revising the manuscript critically.
